# Vasa Previa: Prenatal Diagnosis and the Rationale Behind Using a 5 cm Distance from Internal Os

**DOI:** 10.3390/jcm14031009

**Published:** 2025-02-05

**Authors:** Claudio V. Schenone, Faezeh Aghajani, Ali Javinani, Eyal Krispin, Yinka Oyelese, Ramesha Papanna, Ramen H. Chmait, Alireza A. Shamshirsaz

**Affiliations:** 1 Fetal Care and Surgery Center, Boston Children’s Hospital, Harvard Medical School, Boston, MA 02215, USA; claudio.schenone@childrens.harvard.edu (C.V.S.); faezeh.aghajani@childrens.harvard.edu (F.A.); ali.javinani@childrens.harvard.edu (A.J.); eyal.krispin@childrens.harvard.edu (E.K.); koyelese@bidmc.harvard.edu (Y.O.); 2 Division of Maternal Fetal Medicine, Department of Obstetrics and Gynecology, Beth Israel Deaconess Medical Center, Boston, MA 02215, USA; 3 Department of Obstetrics, Gynecology and Reproductive Sciences, Division of Fetal Intervention, UTHealth McGovern Medical School, Houston, TX 77030, USA; ramesha.papanna@uth.tmc.edu; 4 Department of Obstetrics and Gynecology, Keck School of Medicine, University of Southern California, Los Angeles, CA 90027, USA; ramen.chmait@med.usc.edu

**Keywords:** vasa previa, prenatal diagnosis, distance from internal os

## Abstract

In pregnancies with vasa previa, prenatal diagnosis and pre-labor cesarean delivery are associated with significantly improved perinatal outcomes compared to undetected cases. However, a universally accepted ultrasonographic definition of vasa previa is lacking. Specifically, the distance from the cervical internal os beyond which vaginal delivery can be safely recommended remains to be determined. Field experts and recently published societal guidelines agree that a 2 cm cut-off is suboptimal, given that complete cervical dilation during labor risks unprotected fetal vessels within a 5 cm radius from the internal os. Thus, in the setting of a scarcity of evidence and case reports of perinatal death with unprotected fetal vessels beyond 2 cm from the internal os, a more conservative definition that includes unprotected fetal vessels located within 5 cm of the internal os is imperative to improve outcomes.

## 1. Introduction

Vasa previa is a condition in which fetal arterial or venous vessels, lacking protection from the placenta or Wharton’s jelly, run through the amniotic membranes near or over the internal cervical os [1,2,3,4].

The first case of velamentous cord insertion was observed in 1766 and reported by Wrisberg in 1773 [5,6], and a case of fetal hemorrhage in the setting of velamentous cord insertion was first reported by Lobstein et al. in 1801. Vasa previa was first described in the medical literature by Benckiser in 1831 [3,7]. The pathogenesis of this condition is not fully understood. Placental trophotropism, a phenomenon whereby the placenta grows preferentially toward the more vascularized uterine fundus, may be a contributor. As the less-vascularized placental tissue from the cervix and lower uterine segment undergoes atrophy, exposed fetal vessels may be left behind [8,9].

**Figure 1:**
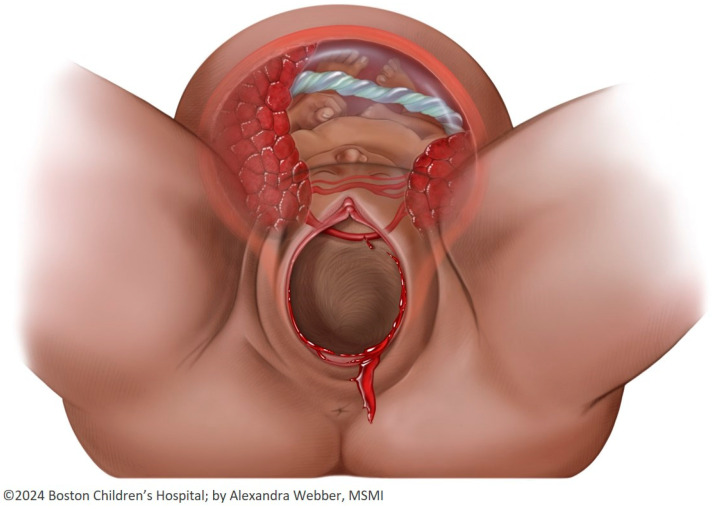
Vasa previa type II.

Vasa previa has been classified into three distinct types according to the vessel’s anatomic configuration. In type I, the cord inserts into the membranes, from where the unprotected vessels traverse over the cervix before reaching the placenta, a condition known as velamentous cord insertion [1,10,11], whereas in type II, they run between the main placental lobe and a succenturiate lobe, also known as an accessory lobe, or in between the two lobes of a bilobate placenta [4,12,13] (Figure 1). More recently, a third type of vasa previa (type III vasa previa) has been described [14,15], in which unprotected fetal vessels left the placental edge unprotected and looped back into the placental margin at a different location. Notably, most of the latter are not accompanied by a velamentous cord insertion or succenturiate placental lobe [16,17,18]. In a case series of fourteen cases of vasa previa published by Kamijo et al., over a third of patients (36%) were classified as being type III vasa previa. They suggested that compared to type I vasa previa, type III vasa previa presents with fewer concomitant risk factors and as a single vessel rather than in pairs [16]. These characteristics, in addition to their course, warrant a high degree of suspicion and thorough evaluation for diagnosis.

This condition poses a significant threat to the fetus. The lack of protection from the Wharton’s jelly or the placental tissue exponentially increases the risk of tearing during labor and spontaneous or artificial rupture of amniotic membranes, resulting in fetal exsanguination (also known as Benckiser hemorrhage) and perinatal death, given that the circulating blood volume in the fetus is only approximately 72 mL/Kg weight at term [19,20,21,22].

Before the advent of prenatal ultrasonography, vasa previa was suspected based on the palpation of fetal vessels within the intact amniotic membrane during a cervical exam, painless vaginal bleeding accompanied by fetal distress (sinusoidal pattern and severe variable decelerations on electronic fetal monitoring), or death [23]. The lack of prenatal confirmation is associated with high perinatal mortality rates. As part of a systematic review and meta-analysis of perinatal outcomes in pregnancies with vasa previa, Zhang et al. reported stillbirth rates of 27.4% and neonatal death rates of 15.8% in 118 pregnancies complicated by vasa previa in whom a prenatal diagnosis was not made; moreover, more than half of the neonates (58%) who survived experienced hypoxic morbidity [24]. Similarly, Oyelese et al. conducted a retrospective review of 155 cases of vasa previa, 94 of whom did not receive a prenatal diagnosis. The perinatal mortality rate in this group was 56% [25].

Gianopolous et al. were the first to report a case of vasa previa detected prenatally using ultrasound in 1987. They described pulsatile loops of cord overlying the cervical os on ultrasound and confirmed their findings at the time of cesarean delivery [26]. Since then, transvaginal ultrasound with color Doppler has become the gold standard for prenatal diagnosis. In a systematic review by Ruiter et al. including 138 cases of vasa previa, transvaginal ultrasound with color Doppler had a median prenatal detection rate of 93% and a specificity between 99 and 100% when performed at 18–26 weeks of gestation [27]. In contrast to undetected cases, elective cesarean delivery before the onset of labor in prenatally diagnosed cases is associated with almost universal perinatal survival. Furthermore, prenatal detection allows for preparation for emergency cesarean delivery to reduce the likelihood of adverse outcomes in the event of vaginal bleeding [28]. Zhang et al. reported a 25-fold and 50-fold reduction in the odds of perinatal mortality and neonatal morbidity, respectively, in cases where vasa previa was diagnosed prenatally, compared to undiagnosed cases [24]. More recently, as part of a systematic review and meta-analysis, Conyers et al. reported a pooled perinatal mortality rate of 0.94% in this patient population. Notably, only about half of the cases of perinatal mortality were directly attributable to ruptured vasa previa [29].

Despite improvements in clinical outcomes with the advent of prenatal diagnosis, a paucity of evidence regarding the optimal distance from the unprotected fetal vessels to the internal os beyond which vaginal delivery can be safely considered for pregnancies with vasa previa remains. This review addresses common misconceptions regarding the definition of vasa previa and the rationale behind adopting a 5-cm distance from internal os as the standard for ultrasonographic diagnosis.

1. **There is a lack of consensus regarding the vasa previa definition regarding distance from internal os**

Although it is clear that the prenatal detection of vasa previa is associated with improved outcomes, a lack of consensus regarding the definition of vasa previa remains (Table 1). In their consult series, the Society of Maternal-Fetal Medicine (SMFM) recognized the lack of standards on the proximity of the fetal vessels to the internal os beyond which vaginal delivery can be safely considered [2]. A 2018 document endorsed by the Nordic Federation of Societies of Obstetrics and Gynecology also recognized the ambivalence between the 2 cm and 5 cm cut-off [30]. The same year, the Royal College of Obstetricians and Gynaecologists explicitly acknowledged the lack of evidence regarding the safe distance from the internal os beyond which vaginal delivery could be considered [31]. Most recently, the Society of Obstetricians and Gynecologists of Canada (SOGC) stated, “There is a lack of consensus regarding the diagnosis of vasa previa” [32]. Additionally, experts in the field continue to raise this issue [33,34,35,36,37,38,39,40,41]. Meanwhile, significant heterogeneity in study definitions and inclusion criteria and wide variations in approaches to care among clinicians remain [42].

2. **There is a lack of evidence regarding a safe distance from the internal os to allow for vaginal delivery in patients with vasa previa**

Many studies assessing perinatal outcomes in patients with vasa previa do not specify a definition according to the unprotected fetal vessel’s distance from the internal os [11,25,35,45]. Catanzarite et al. specified that “the diagnosis of suspected vasa previa was made if the placenta was clear of the internal os of the cervix and fetal vessels were identified over the cervix” [11]. As part of a retrospective multicenter descriptive study, Swank et al. reported that “there were no uniform diagnostic or management approaches because all units function autonomously in clinical decision-making and because none had defined protocols for vasa previa [35]”. In another retrospective review of vasa previa cases, Smorgick et al. defined vasa previa as “parallel or circular echogenic lines “near” the internal cervical os” [45]. Moreover, most of the studies, including a distance from the internal os as part of the ultrasonographic definition, chose 2–3 cm from the internal os as their cut-off, excluding patients in whom the unprotected fetal vessels were beyond this cut-off [33,35,38,46,47].

As part of a retrospective review, Bronsteen et al. reported outcomes of 60 pregnancies with vasa previa, defined as fetal vessels overlying the cervix or in the lower uterus but not directly over the cervix. Among these, there were 14 pregnancies in which the fetal vessels were located up to 3 cm from, but not directly over, the internal os. Two of these cases, in which the fetal vessels were 1.2 cm from the internal os at 27 weeks, required emergent delivery three and four weeks later, respectively, due to vasa previa complications [33].

Poor prenatal outcomes have been reported in the literature for patients who have vasa previa at a distance greater than 2 cm from the internal os. Klahr et al. identified 100 pregnancies complicated by vasa previa, defined as any velamentous fetal vessel (arterial or venous) within 2 cm of the internal cervical os. In this series, vasa previa was deemed resolved (defined as fetal vessels at >2 cm from the internal os during a follow-up scan in their study) in a twin pregnancy at 31 weeks based on a 2.8 cm distance from the internal os at 31 weeks’ gestation. Unfortunately, the patient presented two weeks later with vaginal bleeding, resulting in neonatal death, despite undergoing emergent cesarean delivery [47].

3. **Extrapolating a safe cut-off from pregnancies with low-lying placentas is inappropriate**

Rebarber et al. were the first to use a 2 cm cut-off as the ultrasonographic definition of vasa previa, referencing literature about the likelihood of vaginal bleeding and the need for cesarean delivery according to the distance from internal os in laboring patients with low-lying placentas, proposing that “patients with aberrant velamentous fetal vessels within 2.0 cm of the internal os should be treated like patients with placentas overlying or near the internal os” [34]. A year later, as part of their guidelines, the SMFM stated that “a 2-cm cut-off has been proposed”, referring to the ultrasonographic distance from the internal os for defining vasa previa, citing Rebarber et al. and a clinical expert series article, in which the 2 cm cut-off was mentioned in reference to cases of low-lying placenta, rather than vasa previa [2]. The latter authors raised this issue recently as part of a commentary about misconceptions about vasa previa diagnosis and why a 2 cm distance should not be used as the ultrasonographic definition [40]. Since then, societal guidelines and clinicians have frequently incorporated this cut-off into their institutional protocols and study definitions (Table 1) [2,34,47]. However, complications associated with a low-lying placenta do not pose equivalent risks as those associated with vasa previa. Cesarean delivery may be performed to address bleeding associated with a low-lying placenta during labor without risking the maternal–fetal dyad, given a larger maternal reserve. As part of an observational cohort study, Taga et al. assessed the outcomes of pregnancies with low-lying placentas (defined as a placenta–os distance less than 20 mm) according to the mode of delivery (planned vaginal delivery vs. planned cesarean delivery). There were no differences in the umbilical artery blood pH and frequency of need for oxygen administration or serious neonatal complications between the planned cesarean delivery and the planned vaginal delivery group. Notably, a third of patients in the latter group required emergency cesarean delivery due to uncontrollable antepartum bleeding [9]. Other studies have reported similar results [48,49,50]. In contrast, the rupture of vasa previa and the loss of even small amounts of fetal blood results in catastrophic consequences for the fetus, based on the aforementioned reports on the perinatal outcomes of pregnancies whose vasa previa was not detected prenatally [24,25,29].

4. **Experts agree that a 2 cm definition should not be used**

As a first step toward standardizing the diagnosis and management of pregnancies with vasa previa, and given the lack of randomized controlled trials, our study group recently conducted a four-round focus group discussion and three-round Delphi survey, including a panel of 57 fetal medicine experts spanning thirteen countries, who have contributed to over 80% of published cohort studies on the topic of vasa previa. Consensus was achieved on 26 statements pertaining to the diagnosis and management of vasa previa, one of which was the following: “Although there is no consensus regarding a definition of vasa previa based on distance from the internal os, I feel the definition should not be limited to vessels within 2 cm of the internal os”. Furthermore, when inquired about the definition of vasa previa in their centers, most respondents used definitions other than the 2 cm cut-off. Specifically, 32% of the respondents used a 5 cm cut-off; 21% did not use the distance from the internal os as part of their definition, and 13% used a 2.5–4 cm cut-off [42].

5. **Complete cervical dilation risks fetal vessels within a 5 cm radius from the internal os**

The ultimate goal of the prenatal diagnosis of vasa previa is to prevent unsafe vaginal delivery. The cervix dilates up to 10 cm during labor, placing unprotected fetal vessels within a 5 cm radius of the internal os at risk of rupture as labor progresses (Figure 1). Furthermore, interventions such as rupturing the membranes using an amniotic membrane perforator clearly imperil vessels that are near the cervix [51]. Hence, by using a 2 cm cut-off, a significant proportion of patients with unprotected fetal vessels 2–5 cm from the internal os are exposed to potentially preventable adverse outcomes. In contrast, a 5 cm cut-off may avoid exposing these pregnancies to the risk of vessel rupture and intrapartum or neonatal death.

Societies and experts around the world have raised this issue. The SOGC labels unprotected fetal vessels within 2–5 cm from the internal os as “low-lying fetal vessels”. They recommend considering a cesarean delivery for this patient population, emphasizing the need for careful prenatal assessment and shared decision-making to reduce the risk of adverse outcomes [32,39]. Our study group presented this issue to the Food and Drug Administration (FDA), which accepted the 5 cm cut-off definition as one of the inclusion criteria following extensive review during the pre-recruitment phase of a clinical trial for Fetoscopic Laser Photocoagulation (FLP) in the Management of Vasa Previa (FLUMEN Study) in our center (NCT06290232) [52]. Similarly, other centers offering FLP for pregnancies complicated by vasa previa also include patients in whom the unprotected fetal vessels are located beyond the 2.0 cm cut-off [53,54]. Papanna et al. reported a case undergoing FLP at 32 weeks and 2 days due to vasa previa, whose fetal artery was located 2.7 cm away from the internal os of the cervix. The patient underwent vaginal delivery at 38 weeks and 6 days, and the postnatal course was unremarkable [54].

Adopting a 5 cm distance from internal os as the cut-off for ultrasonographic diagnosis of vasa previa is likely to result in an increased prevalence of this condition and higher rates of preterm pre-labor cesarean delivery and its associated risks in patients who would have otherwise been deemed candidates for a vaginal delivery. However, we believe the risks of perinatal mortality and the potential for long-term neurodevelopmental impairment associated with ruptured vasa previa in such patients outweigh those associated with late preterm cesarean delivery.

This principle also highlights the need to sweep the entire lower uterine segment during transvaginal ultrasound examination to avoid missing unprotected vessels within a 5 cm radius from the internal os that may not be readily apparent in the mid-sagittal view. Ascertaining the entire course of the vessel is paramount to identifying the point at which the distance is closest from the internal os, which may not always be in the mid-sagittal view. This may be achieved by incorporating three-dimensional imaging and other innovative software tools as adjuncts to conventional ultrasound assessment [14,39,40,55,56,57,58,59,60]. Oyelese et al. reported on a case of co-existing placenta and vasa previa detected using 3D sonography, which was not readily apparent on grayscale images. Furthermore, they noted that using three-dimensional images helped map the unprotected vessels to avoid injury at the time of hysterotomy [14]. Lee et al. described using multiplanar views of the cervix and the ‘flight-path’ technique and 3D power Doppler as helpful adjuncts to characterizing the spatial relationship between the unprotected fetal vessels and the internal os of the cervix over 20 years ago [14]. As part of a letter to the editor, Hayata et al. reported on the utility of four-dimensional spatiotemporal image correlation (STIC) for the diagnosis and accurate mapping of vasa previa to avoid injury at the time of hysterotomy [57]. Similarly, Zhang et al. combined STIC with high-definition flow imaging (HD live flow) to obtain an angle-independent enhanced sensitivity to blood flow, which is particularly beneficial for visualizing small vessels, and concluded that this approach allows for the precise observation of the umbilical vessels’ location and their relationship with the placenta. Finally, Gong et al. reported on the use of Flow HD Glass Body from multiple angles to better delineate the relationship between the vasa previa, the placental cord insertion, and the maternal cervix in a three-dimensional plane [58].

Fetal MRI may also help delineate the course of the overlying vessels, especially when the diagnosis is questionable, such as in cases of posterior vasa previa or vasa previa in the setting of bilobed placentas, when sonographic evaluation may be less than adequate [61,62,63,64]. The use of T1-weighted spin echo MRI as an adjunct to prenatal ultrasound for vasa previa diagnosis was first reported by Nimmo et al. in 1987 [61]. Back then, lengthy acquisition times and the need for sedation of the maternal–fetal dyad limited the clinical utility of this imaging modality in obstetrics. Kikuchi et al. reported a case of suspected placenta previa according to ultrasound assessment, in which prenatal MRI demonstrated that the lesion was not placenta but a hemorrhage and that a vessel was running over the internal os freely from the placental tissue [62]. Iwahashi et al. presented the first report of vasa previa in which non-contrast time-of-flight magnetic resonance angiography was used to ascertain the distribution of the fetal vessels [63]. Most recently, as part of a retrospective case series of pregnancies with abnormal placental cord insertions, Tian et al. reported three cases deemed ‘normal’ or ‘unable to visualize’ on ultrasound that received a diagnosis of vasa previa based on MRI assessment [64].

In some cases, fetal head pressure to the lower uterine segment may seem like vasa previa has “resolved”. Kagan et al. reported a case of type II vasa previa that was only able to be visualized when pushing the fetal head upward at the time of follow-up ultrasound examination at 31 weeks’ gestation [41]. In contrast, Oyelese et al. suggested that pushing the fetal head away from the cervix may cause false negative results when using color Doppler to identify the fetal vessel [65]. Therefore, a thorough examination, including repeat assessments in different positions, is warranted before ruling out a resolution.

6. **Distance from internal os should be reassessed at 28–32 weeks and before delivery**

A significant proportion of pregnancies diagnosed with vasa previa during the second trimester no longer meet diagnostic criteria at a subsequent ultrasound examination [38,47,51]. As part of a retrospective study including 165 cases of vasa previa (defined as aberrant vessels within 3 cm of the internal cervical os using grayscale sonography and color Doppler), 43 cases (26%) were deemed “resolved” during the follow-up ultrasound examination. Their reported median and interquartile range for gestational age at resolution was 28.1 weeks (18.4–36.7 weeks). Furthermore, when stratified according to vasa previa type, type I vasa previa was more likely to resolve than type II vasa previa (38.7% vs. 12.5%, *p* = 0.03) [38]. Similarly, as part of a retrospective chart review including 100 vasa previa cases (defined as any velamentous fetal vessel noted to be within 2 cm of the internal cervical os), Klahr et al. reported resolution (defined as fetal vessels > 2 cm from the internal os during a follow-up scan) in 39% of cases. Furthermore, the likelihood of resolution was higher in cases diagnosed at earlier gestational ages. Specifically, they found resolution rates of 66.7%, 40.5%, 26.3%, and 16.7% when diagnosed at <20, 20–24, 24–28, and >28 weeks gestation, respectively. Additionally, unresolved cases had higher rates of vessels directly overlying the internal os (rather than close to but not directly overlying the internal os of the cervix), and they were more likely to have been diagnosed in the setting of resolved placenta previa, compared to cases that resolved. An independent association between diagnosis before 24 weeks, vasa previa not directly overlying the internal os at diagnosis, and vasa previa diagnosis not resulting from a resolved placenta previa and a higher likelihood of resolution upon follow-up ultrasound examination was found [47]. Another retrospective review by Lee et al. included 18 pregnancies complicated by vasa previa, defined as aberrant vessels seen across the internal cervical os, undergoing serial ultrasound examinations to characterize the natural history of this condition. Three of these cases were deemed candidates for vaginal delivery due to the absence of vasa previa, as per the study criteria, during their third-trimester follow-up scan [51]. On the other hand, Hasegawa et al. reported a case of descendent migration of velamentous vessels toward the cervix, resulting in vasa previa [66].

Based on the above, it is essential to reassess the distance from the internal os at 28–32 weeks’ gestation and before delivery in persistent cases to prevent exposing pregnancies that no longer meet the diagnostic criteria to the risks associated with preterm pre-labor cesarean delivery.

## 2. Future Directions

Currently, studies of vasa previa consist almost entirely of cohort studies, case series, and case reports, with wide variations in vasa previa definitions and the frequency of postnatal confirmation using standardized placental pathologic examination. Regarding the latter, RCOG recommends pursuing only in cases of stillbirth or acute fetal compromise, and most other societal guidelines do not make any recommendations in this regard [67]. This is particularly problematic, given a non-insignificant proportion of false positive and false negative cases that would go undetected without routine pathologic examination. As part of a retrospective review, La et al. assessed the accuracy of prenatal ultrasound for diagnosing vasa previa by comparing it with postnatal pathologic examination. They found a 17% rate of false positive cases [68]. Finally, to our knowledge, the majority of vasa previa cohort studies include less than 200 patients, partly due to the relatively low incidence of the condition but also due to a lack of multicentric collaboration [36,38,69].

The aforementioned limitations challenge the ability to ascertain differences in perinatal outcomes according to the unprotected fetal vessel’s distance from the internal os. A randomized trial assessing outcomes in patients with vasa previa according to the mode of delivery while stratifying by fetal vessel’s distance from the internal os is desperately needed to identify the optimal cut-off for ultrasonographic diagnosis, beyond which vaginal delivery could be safely considered. Until such evidence becomes available, and in the setting of the current paucity of evidence and aforementioned reports of perinatal death in cases in which the fetal vessels were beyond the 2 cm cut-off, a more cautious re-examination of the vasa previa definition and using a more conservative cut-off seems prudent. This caution is important given the extremely high perinatal mortality and potential for long-term neurodevelopmental impairment associated with ruptured vasa previa. The extent to which adopting a 5 cm distance from the internal os as the cut-off for prenatal diagnosis will result in a significant difference in the prevalence of this condition, rates of persistence in the third trimester, rates of perinatal death and frequency of preterm pre-labor cesarean delivery is unclear. Thus, further studies are also needed to address these knowledge gaps.

## 3. Conclusions

Transvaginal ultrasound with color Doppler is the gold standard for prenatal diagnosis of vasa previa. Three-dimensional ultrasound assessment, MRI, and other innovative modalities may be helpful as adjunct imaging modalities to ascertain diagnosis in cases where the diagnosis is unclear using grayscale ultrasound. A thorough examination of the entire lower uterine segment is imperative to understanding the relationship between the placenta, placental cord insertion, unprotected fetal vessel, and the internal os of the cervix and identifying vasa previa cases that may not be apparent on mid-sagittal views. When identified during the second trimester, a follow-up examination is warranted at 28–32 weeks, pushing the fetal head away from the lower uterine segment as needed, to rule out cases no longer meeting the criteria for diagnosis. When coupled with preterm pre-labor cesarean delivery in persistent cases, prenatal detection is associated with significantly higher rates of perinatal survival compared to undetected cases.

There is a paucity of evidence regarding the optimal distance from the unprotected fetal vessels to the internal os, beyond which vaginal delivery can be safely considered for pregnancies with vasa previa. The perinatal risks associated with vaginal bleeding during labor are significantly higher in patients with vasa previa compared to those with low-lying placentas. Therefore, extrapolating a safe distance from the internal os from this population of patients with placenta previa to those with vasa previa may be inappropriate. Expert consensus and the most recent societal guidelines agree with the fact that the vasa previa definition should not be limited to unprotected fetal vessels within 2 cm of the internal os of the cervix, given reports of adverse perinatal outcomes in patients with vasa previa at a distance greater than 2 cm from the internal os, and concerns that complete cervical dilation (10 cm) during labor may expose unprotected fetal vessels within a 5 cm radius from the internal os to intrapartum rupture. Therefore, until further evidence arises, a more conservative definition that includes unprotected fetal vessels within 5 cm from the internal os may be prudent.

## Figures and Tables

**Table 1:** Summary of societal ultrasonographic criteria for vasa previa diagnosis.

Society	Year of Publication	Ultrasonographic Criteria	Distance Criteria
Society of Maternal-Fetal Medicine [2]	2015	Arterial vessel within the membranes, with rate consistent with the fetal heart rate, directly overlying the internal os or in close proximity to it, on transvaginal scan	NS
Nordic Federation of Societies of Obstetrics and Gynecology [30]	2018	NS	NS
Royal College of Obstetricians and Gynecologists [31]	2018	Transvaginal color Doppler imaging demonstrating flow and fetal vascular waveforms through at least one aberrant vessel	NS
Royal Australian and New Zealand College of Obstetricians and Gynaecologists [43]	2019	Aberrant linear or tubular echo lucent structures with 2D imaging, with blood flow using color or power Doppler, and umbilical arterial/venous Doppler waveforms using pulsed-wave Doppler on transvaginal ultrasound	2 cm
Society of Obstetricians and Gynaecologists of Canada [32]	2023	Vessels seen running within the membranes above the cervix on transvaginal ultrasound. Fetal heart rate on pulsed-wave Doppler of the artery is helpful but not essential	Vasa previa: <2 cm Low-lying vessel: 2–5 cm
International Society of Ultrasound in Obstetrics and Gynecology [44]	2023	Passage of fetal vessels across or in proximity (usually less than 2 cm) to the internal cervical os using a combination of transabdominal and transvaginal ultrasound	2 cm
Vasa previa in singleton pregnancies: diagnosis and clinical management based on an international expert consensus [42]	2023	NS	No consensus
National College of French Gynecologists and Obstetricians	NA	NA	NA

NS, not specified; NA, not available.

## Data Availability

Not applicable.
